# Involvement of transposable elements in neurogenesis

**DOI:** 10.18699/VJ20.613

**Published:** 2020-03

**Authors:** R.N. Mustafin, E.K. Khusnutdinova

**Affiliations:** Bashkir State Medical University, Ufa, Russia; Institute of Biochemistry and Genetics – Subdivision of the Ufa Federal Research Centre of the Russian Academy of Sciences, Ufa, Russia

**Keywords:** brain, differentiation, noncoding RNA, retroelements, neuronal stem cells, transposable elements, головной мозг, дифференцировка, некодирующие РНК, ретроэлементы, стволовые нервные клетки, транспозоны

## Abstract

The article is about the role of transposons in the regulation of functioning of neuronal stem cells and mature neurons of the human brain. Starting from the first division of the zygote, embryonic development is governed by regular activations of transposable elements, which are necessary for the sequential regulation of the expression of genes specific for each cell type. These processes include differentiation of neuronal stem cells, which requires the finest tuning of expression of neuron genes in various regions of the brain. Therefore, in the hippocampus, the center of human neurogenesis, the highest transposon activity has been identified, which causes somatic mosaicism of cells during the formation of specific brain structures. Similar data were obtained in studies on experimental animals. Mobile genetic elements are the most important sources of long non-coding RNAs that are coexpressed with important brain protein-coding genes. Significant activity of long non-coding RNA was detected in the hippocampus, which confirms the role of transposons in the regulation of brain function. MicroRNAs, many of which arise from transposon transcripts, also play an important role in regulating the differentiation of neuronal stem cells. Therefore, transposons, through their own processed transcripts, take an active part in the epigenetic regulation of differentiation of neurons. The global regulatory role of transposons in the human brain is due to the emergence of protein-coding genes in evolution by their exonization, duplication and domestication. These genes are involved in an epigenetic regulatory network with the participation of transposons, since they contain nucleotide sequences complementary to miRNA and long non-coding RNA formed from transposons. In the memory formation, the role of the exchange of virus-like mRNA with the help of the Arc protein of endogenous retroviruses HERV between neurons has been revealed. A possible mechanism for the implementation of this mechanism may be reverse transcription of mRNA and site-specific insertion into the genome with a regulatory effect on the genes involved in the memory.

## Abstract (ru)

В обзоре представлены накопленные в научной литературе данные об участии мобильных генетических элементов в регуляции дифференцировки нейрональных стволовых клеток и функционирования зрелых нейронов головного мозга. Начиная с первого деления зиготы, эмбриональное развитие управляется закономерными активациями транспозонов, необходимыми для последовательного изменения экспрессии специфических для каждого типа клеток генов. Частным отражением этих процессов может быть дифференцировка нейрональных стволовых клеток – процесс, в ходе которого необходима наиболее тонкая настройка экспрессии генов в нейронах различных областей головного мозга. Доказательствами этого предположения являются данные о высокой активности транспозонов в центре нейрогенеза, зубчатой извилине гиппокампа. Кроме того, мобильные элементы – источники возникновения и эволюции длинных некодирующих РНК, которые коэкспрессируются с необходимыми для работы головного мозга белок-кодирующими генами. Наибольшая активность длинных некодирующих РНК, так же как и транспозонов, обнаружена в центре нейрогенеза человека, что позволяет предположить их участие в управлении работой головного мозга. В регуляции дифференцировкой нейрональных стволовых клеток используются также микроРНК, многие из которых возникают из транскриптов мобильных элементов. Транспозоны посредством собственных процессированных транскриптов играют роль в эпигенетической регуляции дифференцировки нейронов. Объяснением глобальной регуляторной функции мобильных элементов в головном мозге человека может служить их значение в возникновении белок-кодирующих генов в эволюции путем экзонизации, дупликации и доместикации. Эти гены вовлечены в эпигенетическую регуляторную сеть с участием транспозонов, так как содержат нуклеотидные последовательности, комплемен- тарные микроРНК и длинным некодирующим РНК, образуемым из транскриптов мобильных элементов. В формировании памяти выявлена роль обмена вирусоподобными частицами мРНК при помощи белка Arc эндогенных ретровирусов HERV между нейронами. Возможными способами реализации этого механизма могут быть обратная транскрипция мРНК и сайт-специфическая интеграция в геном с регуляторным воздействием на гены, участвующие в консолидации информации.

## Introduction

Transposable elements (TE) make up 69 % of the human genome (de Koning et al., 2011). In the course of evolution, many protein-coding genes (Joly-Lopez, Bureau, 2018), regulatory nucleotide sequences (Ito et al., 2017; Schrader, Schmitz, 2018), and telomeres (Kopera et al., 2011), non-coding RNAs (ncRNAs), including microRNAs (Piriyapongsa et al., 2007; Yuan et al., 2010, 2011; Qin et al., 2015) and long human ncRNAs (Johnson, Guigo, 2014) originating from TE. Over millions of years of evolution, cells have developed various defense systems against TE insertion into their genomes, including DNA methylation, heterochromatin formation, and RNA interference (RNAi). These epigenetic mechanisms have made a significant contribution to the regulation of specific gene expression and cell differentiation (Habibi et al., 2015).

**Fig. 1.**
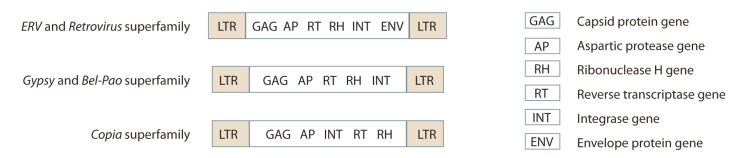
Scheme of the structure of the genes of LTR-containing retroelements.

**Fig. 2.**
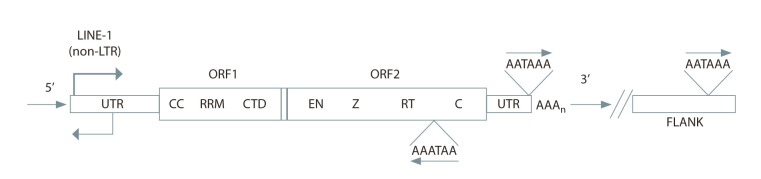
Scheme of the structure of the gene of non-LTR retroelements (LINE-1). UTR – untranslated region; ORF – open reading frame; CC – coiled-coiled; RRM – RNA recognition motif; CTD – С-terminal domain; EN – endonuclease; Z – Z-domain; RT – reverse transcriptase; С – cysteine-rich domain.

**Fig. 3.**
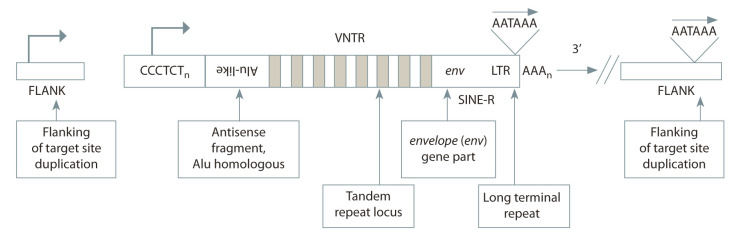
Scheme of the structure of SVA elements.

Transposable elements are divided into two main classes, in accordance with the mechanisms of their transposition. DNA-TEs are transposed by “cut and paste” or “rolling circle”. Retroelements (REs) are integrated into new genome sites using “copy and paste”. REs are classified into those containing long terminal repeats (LTR REs) (Fig. 1) and those not containing them (non-LTR REs) (Fig. 2). The latter are divided into autonomous (LINE, long interspersed nuclear elements) and non-autonomous (SINE, short interspersed nuclear elements) and SVA (SINE-VNTR-Alu) (Fig. 3) (Klein, O’Neill, 2018).

The human genome contains more than 500,000 copies of LINE1 (L1), which make up 17 % of all nucleotide sequences. Among them, only about 100 L1 are active, since they contain the full length of 6000 bp. Among non-autonomous REs, the human genome contains more than 2700 copies of SVA (Hancks, Kazazian, 2012). One of the important factors for the development of the human brain is considered the waves of L1 retrotranspositions, as well as the birth of new TEs, such as SINE, Alu and SVA in the evolution of primates (Linker et al., 2017).

Human endogenous retroviruses (HERV) belong to LTRRE. They occupy about 8 % of the entire genome and serve as sources of a huge number (794,972) of binding sites with specific transcription factors (TFs), the activation of which plays a role in embryogenesis. For example, in the mesoderm, LTRs interact with SOX17, FOXA1, GATA4; in pluripotent cells, with SOX2, NANOG, POU5F1; in hematopoietic cells, with TAL1, GATA1, PU1 (Ito et al., 2017). Mammalian-wide interspersed repeats (MIRs), which belong to the ancient SINE family descended from tRNA, are also associated with tissuespecific gene expression (Jjingo et al., 2014).

Transposable elements are characterized by nonrandom activation, depending on the tissue and stage of development. High-throughput profiling of integration sites by nextgeneration sequencing, combined with large-scale genomic data mining and cellular or biochemical approaches, has revealed that the insertions are usually non-random (Sultana et al., 2017). Programmed activation of TE in individual cells during neurogenesis leads to a change in the expression of certain genes necessary for differentiation into specific types of neurons for the formation and functioning of brain structures (Coufal et al., 2009; Bailie et al., 2011; Thomas, Muotri, 2012; Richardson et al., 2014; Evrony et al., 2015; Upton et al., 2015; Muotri, 2016; Suarez et al., 2018). In accordance with this, somatic mosaicism of neurons detected by insertions of TEs (Richardson et al., 2014; Upton et al., 2015; Bachiller et al., 2017; Paquola et al., 2017; Rohrback et al., 2018; Suarez et al., 2018) can reflect the programmed regulatory pattern of the genome necessary for the maturation of specific structures of the central nervous system (Paquola et al., 2017; Rohrback et al., 2018). Somatic mosaicism means the presence, in the same organism, of cells with different genomes as a result of de novo DNA changes. These structural variations may be due to CNV, insertions of REs, deletions under the influence of TEs, and SNV (Paquola et al., 2017). This means that in different cells of one organism, not only the genotype, but also the whole genome changes. This is due to the occurrence of mutations in exons of protein-coding genes, intergenic regulatory regions and introns, which is accompanied by a specific expression of certain genes specific for each cell type.

## The role of transposable elements in neuronal differentiation

The human brain contains an average of 86.1 billion neurons. Moreover, each of the neurons forms from 5,000 to 20,000 synaptic connections, creating a complex network with a variety of cell types and subtypes. The number of subtypes of neurons is so large that it does not lend itself to modern methods for their description. There must be mechanisms to ensure such a diversity of neurons with their specific temporal and spatial features of functioning (Thomas, Muotri, 2012). The sources of these mechanisms can be TEs, combinations of movements of which can become sources of countless variety. An example of this is the molecular mechanism for generating antibodies by the mammalian immune system (V(D)J recombination), derived from TEs (Lapp, Hunter, 2016). TEs played a role in the development of the central nervous system. In evolution, they turned out to be sources of the formation of regulatory structures and genes involved in the formation of the brain. Non-autonomous TEs MER130 were preserved in the genomes during evolution due to their location near the neocortex genes as a necessary link for their regulation. The experiments showed the activation of MER130 in mouse embryos on the fourteenth day of development as gene enhancers for the development of the neocortex (Notwell et al., 2015). Among 11 sushi-ichi-specific placental animal genes derived from REs, the SIRH11/ZCCHC16 gene encoding zinc finger CCHC protein contributed to the evolution of the brain. This domesticated gene is involved in the development of cognitive functions of placental animals (Irie et al., 2016).

In 2009, in neuronal stem cells isolated from the brain of a human embryo, L1s retrotranspositions were detected, as well as an increase (in comparison with the liver and heart of the same individual) in the number of copies of endogenous L1s in the adult hippocampus (Coufal et al., 2009). In addition to L1 (7743 insertions), a large number of somatic transpositions Alu (13,692 insertions) and SVA (1350 insertions) were found in the hippocampus of adults (Bailie et al., 2011). These de novo integrations can affect the expression of certain genes, creating unique transcriptomes of individual neurons (Muotri, 2016). This may be due to the genome-programmed TE ability for their regular site-specific insertions (Sultana et al., 2017). In 2009, of 19 retrotranspositions, 16 were found at a distance of less than 100 kilobases from genes expressed in neurons (Coufal et al., 2009). In 2015, in a study of the somatic mosaicism of the human hippocampus K.R. Upton et al. revealed, out of 20 identified L1 transpositions, 2 functionally significant insertions into the introns of the ZFAND3 and USP33 genes functioning in the brain (Upton et al., 2015). A.A. Kurnosov et al., when studying human brain samples, showed that out of 3100 transpositions of L1 in neurons of the dentate gyrus of the hippocampus, 50.26 % of insertions are located in the genes, and out of 2984 Alu, 49.1 % (Kurnosov et al., 2015). In 2016, J.A. Erwin et al. revealed that in the brain of healthy people 44–63 % of neurons undergo somatic mosaicism at the loci of genes that are important for the functioning of the nervous system. For example, a high insertion frequency of L1-RE is shown for the DLG2 gene, which affects cognitive flexibility, attention, and learning. Mutations in DLG2 are associated with the development of schizophrenia (Erwin et al., 2016).

Somatic retrotranspositions, unlike germinal ones, cannot be inherited by future generations. However, the programmed ability for specific insertions, depending on the composition and location of TEs in the genome, can be inherited. An explanation of the ability of TEs to be inserted in a site-specific manner in the region of genes expressed in the brain may be the evolutionary relationship of protein-coding genes and their regulatory sequences with TEs (Gianfrancesco et al., 2017; Ito et al., 2017; Joly-Lopez, Bureau, 2018). The insertions specific for humans and chimpanzees were revealed near the promoters of the tachycin receptor genes TACR3, cation channels TRPV1 and TRPV3, oxytocin OXT. These genes are associated with the functioning of neuropeptides. Analysis of the genomes of various mammals showed that the neural enhancer nPE2, which regulates the expression of the POMC gene in the hypothalamus, evolved from SINE in evolution (Gianfrancesco et al., 2017).

Transpositions and expression of TEs can vary depending on the area of the brain and change under environmental influences, as they can perform a number of adaptive functions (Lapp, Hunter, 2016). More active are L1, which retained the ability to transpose, causing somatic mosaicism (Suarez et al., 2018). In 2005, A.R. Muotri et al. suggested that L1 using somatic transpositions can actively create mosaicism of neuron genomes (Muotri et al., 2005). In the brain, somatic mosaicism plays an important role in the regulation of cognition and behavior. The consequences of somatic mosaicism encompass vast changes – from a variant at a single locus, to genes in neuronal networks (Paquola et al., 2017; Rohrback et al., 2018). Moreover, the features of somatic mosaicism differ between neurons of various regions of the brain. For example, in the cerebral cortex, only 0.6 insertions of L1-RE are observed, while in the hippocampus, from 80 to 800 inserts per neuron (Lapp, Hunter, 2016). Somatic mosaicism due to retrotranspositions is a source of phenotypic diversity between neurons during development. In the brain of an adult under the influence of various environmental factors, L1 expression can affect the functioning of neurons during the formation of long-term memory (Bachiller et al., 2017).

The hippocampus is the center of human neurogenesis, where many insertions affect transcriptional expression, creating unique transcriptomes in neurons. In addition, transcriptional activation of L1 is similar to that for the NeuroD1 gene. This may indicate the effect of L1 expression on neurogenesis, since stimulation of Wnt3a in neuronal stem cells increases L1 expression 10-fold along the beta-catenin pathway, similarly activating transcription of the NeuroD1 gene. This gene encodes the transcription factor that activates the genes involved in neurogenesis. The NeuroD1 promoter region contains a Sox/LEF site similar to the 5ʹUTR of the L1 element, and the pattern of time expression of the NeuroD1 and L1 genes during differentiation of neurons is similar (Thomas, Muotri, 2012).

Genetic variations between neurons due to L1 retrotranspositions may be associated with specific enrichment of neuronal stem cell enhancers. It was shown that specific enhancers for certain types of neurons (determined using FANTOM5) correspond to the coordinates in the genome for insertions L1, which are within 100 bp from the enhancer. These patterns have not been identified for astrocytes and hepatocytes (Upton et al., 2015). When studying the features of L1 retrotranspositions in more than 30 regions of the brain, a lot of L1 insertion-specific cell lines were found (Evrony et al., 2015). In experiments on mice, specific L1 expression was also shown depending on the area of the brain and the age of the animal (Cappucci et al., 2018).

In addition to L1 elements, LTR-REs are also involved in the regulation of neurogenesis. For example, in mice, the region where the full-length ERVmch8 on chromosome 8 was located was comparatively less methylated in the cerebellum, due to its specific expression depending on the stage of development (Lee et al., 2011). In accordance with these data, it can be assumed that the features of TEs activation observed in neuronal stem cells can naturally alter the expression of specific genes necessary for differentiation of neurons during the formation of specific brain structures. The reason for the activation of TEs in the neuronal stem cells of the hippocampus and the reason for their importance in memory consolidation may be the sensitivity of TEs to stressful environmental influences (Mustafin, Khusnutdinova, 2019). These mechanisms are a particular reflection of the general pattern of epigenetic control of the development of the whole organism, starting from the first division of the zygote, under the regulatory influence of TEs (Mustafin, Khusnutdinova, 2018). To understand the role of TEs in these processes, it is necessary to consider their participation in embryogenesis.

## The role of transposable elements in embryogenesis

To initiate the development of the body after fertilization, gametes are reprogrammed to totipotency. During this reprogramming, TEs activation is observed. Previously, this phenomenon was believed to be a side effect of extensive chromatin remodeling at the basis of epigenetic reprogramming of gametes. However, a targeted epigenomic approach has been performed to determine whether TEs directly affect chromatin organization and body development. It was found that silencing of L1 elements reduces the availability of chromatin, and prolonged activation of L1 prevents its gradual compaction, which occurs naturally during development. That is, L1 activation is an integral part of the development program (Jachowicz et al., 2017). In experiments on mice, the role of LTR-REs as a necessary control element for early embryogenesis was proved (Wang et al., 2016). 

For the cis-regulatory activity of the LTR retroelements ERVK, MERVL and GLN, a complex of RNA and proteins is required, formed using the long ncRNA LincGET. Artificial silencing of LincGET expression in the embryo at the bicellular stage leads to a complete halt to further development due to disruption of cis-regulation of the genes necessary for proliferation under the influence of LTR-REs driven by LincGET (Wang et al., 2016). It has also been shown that HERVs are activated in all types of human cells with characteristic features for certain tissues and organs (Seifarth et al., 2005). In the study of the association of 112 TE families in 24 human tissues, tissue-specific enrichment of active regions of LTR-REs was noted, which indicates the involvement of LTE-REs in the regulation of gene expression for differentiation of cells depending on their functional purpose in ontogenesis. This is due to the presence, in the TEs sequences, of transcription factors binding sites (TFBSs) that regulate the development of the corresponding tissue. TE enrichment characteristic of certain cells in intron enhancers correlates with tissue-specific variations in the expression of nearby genes (Trizzino et al., 2018).

The genetic program in the 2-cell stage of embryogenesis in mice and humans is largely controlled by transcription factors of the DUX family, which are key inducers of zygote genome activation in placental mammals (De Laco et al., 2017). L1 transcripts in embryos are necessary for Dux silencing, rRNA synthesis and exit from the 2-cell stage. M. Percharde et al. in their article showed that L1 expression is required for preimplantation development (Percharde et al., 2018). In embryonic cells, L1 transcripts act as a nuclear RNA scaffold that recruits Nucleolin and Kap1/Trim28 factors for Dux repression. In parallel, L1 products mediate the binding of Nucleolin and Kap1 to rDNA, contributing to the synthesis of rRNA and self-renewal of embryonic stem cells (Percharde et al., 2018). The role of L1 in the repression of the transcriptional program of a 2-cell embryo indicates their participation in the development-specific regulation of gene expression necessary for cell differentiation and body development (Jachowicz et al., 2017). It can be assumed that the activity of REs in neuronal stem cells indicates their use as switches of transcription programs in the specific functionalization of neurons. That is, TEs are involved in the management of both the differentiation of embryonic cells and postnatal stem cells. Regulation is carried out by implementing information encoded in the features of the composition and distribution of TEs in the genome, through the sequential activation of strictly defined TEs in each new cell, specific for the tissue and stage of development. The greatest role is played by this species-specific “coding” in the brain, where neurons are distinguished by higher activity of REs. This is reflected in the structural and functional complexity of the brain compared to other organs. The use of TEs as sources of ncRNAs plays an important role in these processes.

## The relationship of transposable elements with non-coding RNAs in the brain

According to recent data, from 75 to 85 % of the human genome is transcribed into primary transcripts, while only 1.2 % of the genome is translated into proteins. Most transcripts are registered as ncRNAs that are involved in the regulation of the genome (Djebali et al., 2012). In humans, 13,000 genes of long ncRNAs have been identified, for the occurrence of which HERVs are responsible by insertion of promoters. HERV-stimulated long ncRNAs are characterized by specific transcription in different types of pluripotent cells, which is consistent with the over-expression of these HERVs in human embryonic stem cells (Johnson, Guigo, 2014). Transcription of most long ncRNAs is associated with the expression of protein coding genes according to the type of neurons and a specific region of the brain. For example, according to Allen Brain Atlas in situ hybridization data, out of 1328 known long mouse ncRNAs, 849 are expressed in their brain and are associated with cell types and subcellular structures. The biological significance of these ncRNAs in the functioning of neurons and their relationship with protein-coding genes has been shown (Mercer et al., 2008).

Long ncRNAs expressed in the brain, such as Miat, Rmst, Gm17566, Gm14207, Gm16758, 2610307P16Rik, C230034O21Rik, 9930014A18Rik, share a similar expression model with neurogenesis genes and overlap these genes, which proves the role of long ncRNAs in neurogenesis (Aprea et al., 2013). These data are consistent with the role of TEs in neurogenesis (Coufal et al., 2009; Kurnosov et al., 2015; Erwin et al., 2016; Muotri, 2016) and regulation of brain function (Thomas, Muotri, 2012; Upton et al., 2015; Rohrback et al., 2018). This is because TEs are the main sources of the emergence and evolution of long ncRNAs, forming their functional domains and making up more than 2/3 of their mature transcripts in humans (Kapusta, Feschotte, 2014). REs can serve as genes for long ncRNAs (Lu et al., 2014). L1s have a function similar to lncRNA in regulating the expression of genes necessary for self-renewal of stem cells and for preimplantation development (Honson, Macfarlan, 2018).

In a number of studies, the role of miRNAs in controlling the differentiation of neurons, switching expression profiles of genes important for cell function in time and space has been proved (Stappert et al., 2015). About 40 % of all known human miRNAs are expressed in the human brain. The specific expression of many of them differs in different types of cells and is important in the regulation of differentiation, which is necessary for a huge variety of phenotypes of neurons in the brain (Smirnova et al., 2005). The accumulation of certain miRNAs in various structures of neurons (axons, dendrites, synapses) was revealed. For example, in experiments in mice, the role of miR-134 in the regulation of specific mRNAs of the LIMK1 gene for the growth of dendritic spins was shown, and the accumulation of miR-99a, 124a1-3, 125b1, 125b2, 134, 339 was noted in synaptosomes (Lugli et al., 2008). The formation of neurites is promoted by miR-21 (the target is the mRNA of the SPRY2 gene), miR-431 is involved in the regeneration of axons (the target is the Kremen-1 gene), differentiation of neurons occurs under the influence of miR- 34a (the targets are Tap73, synaptotagmin-1, syntaxin-1A) and miR-137 (targets are the Mib1, Ezh2 genes). Enhanced expression of miR-9 promotes branching and reduced axon growth by repressing microtubule-associated Map1b protein. Axon growth depends on the effect of miR-431, as well as miR-17-92, which interacts with PTEN (phosphate tensin homolog) in neurons of the cerebral cortex of the embryo. The regulatory role of differential expression of miR-221 and miR-222 in neurogenesis has also been proven (Nampoothiri, Rajanikant, 2017).

**Fig. 4.**
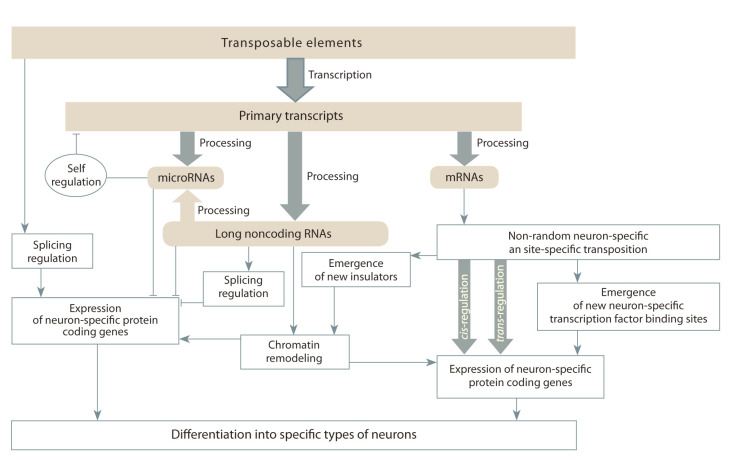
Scheme of TE involvement in neurogenesis.

In 2007, J. Piriyapongsa et al. found that in humans TEs can be sources of microRNAs (Piriyapongsa et al., 2007), which was confirmed by other researchers (Yuan et al., 2010, 2011; Qin et al., 2015). The key role of TEs in the formation of microRNAs and long ncRNAs (Johnson, Guigo, 2014; Ka-pusta, Feschotte, 2014) indicates that the maximum activity of TEs at the center of human neurogenesis (Kurnosov et al., 2015) as a natural phenomenon is necessary for epigenetic control of differentiation of neuronal stem cells. Another mechanism of TE participation in the regulation of gene expression necessary for the specific work of neurons is the cisand trans-effects of TEs (Garcia-Perez et al., 2016). This confirms the nonrandom activations of TEs as sources of heterogeneous subpopulations of neurons (Fig. 4) (Faulkner, 2011).

## The role of retroelements in interactions between neurons

For the development and functioning of the brain, intercellular interactions are necessary, the study of the regulation mechanisms of which is promising for therapeutic targeted exposure to the work of the brain. For this, it is important to identify drivers for gene expression and post-transcriptional epigenetic regulation of the structural components of neurons. Based on the analysis of the accumulated data on the role of TEs in controlling the functioning of the genome in embryonic development (Garcia-Perez et al., 2007; Van den Hurk et al., 2007; Macia et al., 2011; Kurnosov et al., 2015; Percharde et al., 2018) and the physiological functioning of the human brain (Coufal et al., 2009; Bailie et al., 2011; Thomas, Muotri, 2012; Richardson et al., 2014; Evrony et al., 2015; Upton et al., 2015; Muotri, 2016; Suarez et al., 2018), it was concluded that TEs are regulators of epigenetic control for gene function in ontogenesis (Mustafin, Khusnutdinova, 2017, 2018). Despite the lack of mitotic activity of mature neurons, the specific expression of TEs in them is important in controlling both interneuronal interactions and the structural and functional characteristics of neurons (Bailie et al., 2011; Richardson et al., 2014; Erwin et al., 2016). These properties may be due to processing from transcripts of transposons of specific long ncRNAs (Lu et al., 2014; Honson, Macfarlan, 2018) and microRNAs (Piriyapongsa et al., 2007; Yuan et al., 2010, 2011; Qin et al., 2015). Indeed, in experiments on laboratory animals, the enrichment of specific miRNAs in certain structures and regions of neurons was revealed. For example, an abundance of miR-15b, miR-16, miR-204, miR-221 was found in the distal axons compared to neuron bodies (Natera-Naranjo et al., 2010). Enrichment of specific miRNAs in synapses was detected. This suggests a local post-transcriptional regulation of the expression of neuron-specific genes (Lugli et al., 2008). The role of miRNAs in intercellular interactions in the brain was shown, as well as the value of the electrical activity of neurons for the secretion of miR-124 and miR-9, which can penetrate microglia and change the phenotype of its cells (Veremeyko et al., 2019).

Transposable elements regulate brain function through expression into specific microRNAs that regulate gene expression in neurons and in intercellular interactions in the brain. The role of ERV in transferring information between neurons for memory consolidation has also been identified. In the human genome, the full-length HERV-K (about 10,000 bp) consists of the remains of ancient retroviruses and includes LTR-flanked regions, including three retroviral ORFs: pol-pro (encodes protease, RT and integrase enzymes), env (encodes horizontal transfer proteins) and gag (encodes structural proteins of the retroviral capsid) (Klein, O’Neill, 2018). In the course of evolution, the specific ERV Ty3/gypsy has become the source of Arc protein. This protein is similar in biological properties to the gag retroviral gene expression product (Pastuzyn et al., 2018).

Since domestication and use for the needs of the host, the Arc gene has become highly conserved for vertebrates, playing a role in the functioning of their brain. Expression of Arc is highly dynamic in the brain in accordance with the encoding of information in neural networks. Arc gene transcript is transported to dendrites and accumulates in areas of local synaptic activity, where translation into protein occurs (Shepherd, 2018). In neurons, the Arc protein forms spatial structures resembling viral capsids that encapsulate cell mRNA. The resulting virus-like elements in the composition of extracellular vesicles are transmitted to neighboring neurons, where they are able to translate. This phenomenon is used to consolidate long-term memory (Pastuzyn et al., 2018), in the formation of which the hippocampus is involved, where the maximum activity of TEs is detected (Coufal et al., 2009; Bailie et al., 2011; Thomas, Muotri, 2012; Bachiller et al., 2017).

Based on the data listed above, it can be concluded that the observed phenomenon of intercellular neuronal interconnection using Arc has developed in evolution as a reflection of the adaptive value of the TE transcript transfer phenomenon between postmitotic cells. It is possible that when neurons exchange virus-like mRNA particles between neurons, the ability of TEs to be integrated in a site-specific manner (Sultana et al., 2017) with a change in the expression of neuron-specific genes is used to form long-term memory. As a result, the functioning of neurons and the storage of information in the brain change (Bachiller et al., 2017).

## Other functions of transposable elements

Transposable elements transpositions affect gene expression in various ways. Insertions within a gene can cause frameshift mutations, premature stop codons, or exon skipping. In the transcribed portion of the gene, TEs can reduce mRNA levels by slowing transcription due to the high A/T content in ORF2 of TEs such as L1 RE (Thomas, Muotri, 2012). However, despite the potentially mutagenic effect TEs play a role in the evolution of the genomes of all eukaryotes through the use of TE sequences to form host adaptive abilities (Mustafin, Khusnutdinova, 2019). TEs are involved in controlling the expression of protein-coding genes, many of which (Joly-Lopez, Bureau, 2018), including transcription factors (Ito et al., 2017), originated from TEs. In addition to the direct domestication of TEs, new protein-coding genes were formed due to exonization and duplication of genes using TEs (Thomas, Muotri, 2012; Joly-Lopez, Bureau, 2018; Mustafin, Khusnutdinova, 2018).

Mechanisms derived from TEs are used by the mammalian immune system to generate antibodies using the V(D)J recombination system. TEs are the source of most steroid receptors, participating in the global regulation of cell function by the hormonal system (Lapp, Hunter, 2016). Regulatory sequences, silencers, and insulators evolved from TEs (Jjingo et al., 2014; Ito et al., 2017; Schrader, Schmitz, 2018). If TEs are inserted into non-coding regions of genomes, they are used as alternative promoters, enhancers, and polyadenylation signals of genes. For example, L1s are found in non-coding regions of 80 % of human genes, the expression pattern of which depends on the density of these REs (Klein, O’Neill, 2018).

About 60 % of all SVAs in the human genome are located in the genes or flank them within 10 kb. These SVAs are characterized as mobile CpG islands capable of upstream or downstream regulation of gene expression by recruiting transcription factors. In addition, due to the high GC content, SVAs can form alternative DNA structures, such as the G- quadruplex (characteristic of promoters of 40 % of human genes), which affects transcription (Gianfrancesco et al., 2017). Many transcription factors are immediately directed to the relationship with TEs, forming and maintaining heterochromatin (Lapp, Hunter, 2016). TEs serve as sources of cis- and transregulatory elements that coordinate the expression of groups of genes. In addition to acting as promoters that control the expression of alternative host gene isoforms, TFBS within TEs can act as enhancers in certain tissues and at certain stages of development (Garcia-Perez et al., 2016).

In evolution, ТЕs were the sources of a significant part of the specific sequences of the genome, as well as transcripts and proteins interacting with them. This indicates a global regulatory role of TEs, necessary for both mitosis and meiosis, and for controlling the work of cells in interphase. For example, not only spliceosomal introns (Kubiak, Makalowska, 2017), but also the Prp8 spliceosome component originated from TEs (Galej et al., 2013). Splicing enhancers and silencers are 10-nucleotide-long ncRNAs that interact with SR proteins and snRNAs. They are formed by processing transcripts of Alu retroelements (Pastor et al., 2009). TEs turned out to be sources of satellites due to the capability of site-specific insertions (McGurk, Barbash, 2018) and illegitimate recombination, followed by amplification by gene conversion (Han et al., 2016). In evolution, TEs have become sources of telomerase and telomeres (Kopera et al., 2011), as well as centromeres (Cheng, Murata, 2003; Sharma et al., 2013; Han et al., 2016) and the protein CENP/CENH3 interacting with them (Lo-pez- Flores et al., 2004; Volff, 2006). Small ncRNAs formed upon transcription of centromeric REs are involved in the regulation of these interactions (Carone et al., 2013).

## Conclusion

Less than 1.2 % of the human genome is responsible for the coding of proteins. The remaining non-coding part of the genome was largely formed due to TEs. The data on the participation of TEs in the regulation of gene switching during cell differentiation in embryogenesis, starting with the first zygote division, suggest that somatic mosaicism observed in neurons reflects the active role of TEs in neurogenesis. A number of papers have been published proving the participation of TEs in the control of differentiation of neurons. Transposable elements are sources of ncRNA, which are also important in gene switching in brain cells. The revealed role of LTRcontaining REs in the exchange of transcripts between neurons may reflect the general principle of the participation of TEs in the regulation of gene expression for the development and maintenance of brain function. The use of Arc protein for the formation of virus-like particles in the transfer of information between cells indicates the evolutionary mechanisms of TE conversion into viruses for the formation of adaptive functions. This mechanism is associated with the use of TEs to ensure the dynamism of the genomes of postmitotic cells with the possibility of their adaptive changes in response to environmental influences. The realization of this phenomenon is possible due to reverse transcription of mRNA transported between cells with site-specific insertions, the formation of somatic mosaicism of mature neurons, and a change in gene expression for memory consolidation.

## Conflict of interest

The authors declare no conflict of interest.

Since somatic mosaicism cannot be inherited, the functional role of TE insertions in neurogenesis is difficult to prove. Moreover, these changes can be characterized as random events that are more important for the development of neurological disorders. However, the data presented in the review prove the importance of TE transpositions into functionally significant regions of the genome, which are necessary for differentiation of neuronal stem cells and the response to environmental influences. The explanation of this regular phenomenon is the capability of TEs to be inserted in a site-specific manner programmed by their own position in the genome. These nonrandom events are selected during the evolution of multicellular organisms, promoting regulatory regulation of gene expression during cell differentiation.

The results obtained on the importance of TE transpositions in neurogenesis reflect one of the stages of regulation of gene expression in successive cell divisions during differentiation of tissues and organs of the whole organism. Somatic mosaicism in neurons and stem cells is in favor of this assumption, since the brain is characterized by a pronounced variety of cell types, for the specific tuning of gene expression of which universal combinatorial units, such as TEs, are required.
